# A novel analytical method, Birth Date Selection Mapping, detects response of the Angus (*Bos taurus*) genome to selection on complex traits

**DOI:** 10.1186/1471-2164-13-606

**Published:** 2012-11-09

**Authors:** Jared E Decker, Daniel A Vasco, Stephanie D McKay, Matthew C McClure, Megan M Rolf, JaeWoo Kim, Sally L Northcutt, Stewart Bauck, Brent W Woodward, Robert D Schnabel, Jeremy F Taylor

**Affiliations:** 1 Division of Animal Sciences, University of Missouri, Columbia, MO, 65211, USA; 2 Department of Biology, Duke University, Durham, NC, 27708, USA; 3 Department of Animal Science, University of Vermont, Burlington, VT, 05405, USA; 4 Bovine Functional Genomics Laboratory, ARS, USDA, Beltsville, MD, 20705, USA; 5 Department of Animal Science, Oklahoma State University, Stillwater, OK, 74078, USA; 6 American Angus Association, 3201 Frederick Ave, Saint Joseph, MO, 64506, USA; 7 GeneSeek, 4665 Innovation Drive, Suite 120, Lincoln, NE, 68521, USA; 8 NextGen, Duluth, GA, 30096, USA

**Keywords:** Selective sweep, Selection mapping, Polygenic model, Pleiotropy, Cattle, Phenotype, Genotype, GWAS, SNP

## Abstract

**Background:**

Several methods have recently been developed to identify regions of the genome that have been exposed to strong selection. However, recent theoretical and empirical work suggests that polygenic models are required to identify the genomic regions that are more moderately responding to ongoing selection on complex traits. We examine the effects of multi-trait selection on the genome of a population of US registered Angus beef cattle born over a 50-year period representing approximately 10 generations of selection. We present results from the application of a quantitative genetic model, called Birth Date Selection Mapping, to identify signatures of recent ongoing selection.

**Results:**

We show that US Angus cattle have been systematically selected to alter their mean additive genetic merit for most of the 16 production traits routinely recorded by breeders. Using Birth Date Selection Mapping, we estimate the time-dependency of allele frequency for 44,817 SNP loci using genomic best linear unbiased prediction, generalized least squares, and BayesCπ analyses. Finally, we reconstruct the primary phenotypes that have historically been exposed to selection from a genome-wide analysis of the 16 production traits and gene ontology enrichment analysis.

**Conclusions:**

We demonstrate that Birth Date Selection Mapping utilizing mixed models corrects for time-dependent pedigree sampling effects that lead to spurious SNP associations and reveals genomic signatures of ongoing selection on complex traits. Because multiple traits have historically been selected in concert and most quantitative trait loci have small effects, selection has incrementally altered allele frequencies throughout the genome. Two quantitative trait loci of large effect were not the most strongly selected of the loci due to their antagonistic pleiotropic effects on strongly selected phenotypes. Birth Date Selection Mapping may readily be extended to temporally-stratified human or model organism populations.

## Background

Several statistical tests have been developed to identify the genomic regions that have been subjected to strong recurrent selection. Most have been based on extreme population differentiation [1-3], the enrichment of rare mutations in the site frequency spectrum [4,5], or patterns of extended haplotype homozygosity [6-8] (See [9,10] for further review). These tests have now been used to detect molecular signatures of selection in cattle [11-16]. However, recently, there has been a call to employ polygenic models to simultaneously identify loci responding to selection but that do not fit the typical “hard sweep” paradigm [17,18].

Concurrent with the development of new approaches for the detection of selective sweeps, the statistical models employed for genome-wide association studies have been improved. Some of the refinements deal with the effects of population structure and kinship between sampled individuals, since not accounting for these effects can significantly increase the number of false positive associations (See [19] for review). It has been shown that fitting a genomic relationship matrix (also known as a kinship matrix) effectively prevents false positives due to population structure and kinship [19,20]. Furthermore, there has also been a shift toward the application of polygenic models for the identification of genetic risk factors and variants associated with complex phenotypes [21,22].

In this study, we merge the search for loci responding to selection with advanced genome-wide association models to quantify the genome-wide response to selection in US registered Angus cattle. We introduce a novel method, Birth Date Selection Mapping, for identifying loci that are responding to ongoing selection.

Selection induces changes in allele frequency for the selected mutation, as well as for neighbouring loci that hitchhike along with the selected locus due to the presence of linkage disequilibrium between the loci. Accordingly, individual allele counts (0, 1, or 2 for *AA*, *AB*, and *BB* genotypes) could be regressed on birth date using a Poisson model to identify loci that have rapidly changed in frequency over time. However, in the presence of any sampling bias (non-random ascertainment of family members in time, population structure, or kinship), this approach suffers from a very high false-positive rate of detection of loci subject to selection (Additional file 1: Figures S1 and S2). The bias results from a pedigree-based stratification in the depth of sampling of DNA on individuals within different families and differences in allele frequencies between families such that the differences in allele frequencies between families are partially confounded with differences in allele frequencies in time. In other words, this approach is confounded by pedigree relationships and the non-random sampling of individuals from families at different time points. The use of a mixed linear model with allele counts or frequencies fit as the dependent variable and a random animal term fit using a numerator or genomic relationship matrix does not solve the problem because any time-dependent trend in allele frequency is now incorporated into the solutions for animal effects (data not shown).

In our approach, rather than regressing allele frequencies (dependent variable) on birth date (explanatory variable), we invert the relationship and fit birth date as the dependent variable and identify SNPs that are strongly associated or predictive of birth date. If a neutral DNA variant is drifting through a population, changes in allele frequency will be stochastic and small provided the effective population size (*N*_*e*_) is large. For these variants, the probability of a specific genotype will be approximately constant in time and knowledge of an individual’s genotype will not be strongly predictive of that individual’s birth date. On the other hand, if a variant is under strong directional selection, changes in allele frequency will be consistent in direction and may become large over several generations. For these variants, genotype will be predictive of birth date. For example, if the *A* allele is increasing in frequency in time, *AA* individuals are much more likely to be born recently than in the distant past. We utilize mixed model methods to account for the pedigree-based structure in our sample through the use of the genomic relationship matrix [19-21]. With the analysis framed from this perspective, we identify the SNPs that are changing in frequency due to selection while accounting for kinship within the sample. By so doing, we are the first to apply polygenic models for the detection of genomic imprints of selection.

## Results

### Evidence of selection

**Figure 1:**
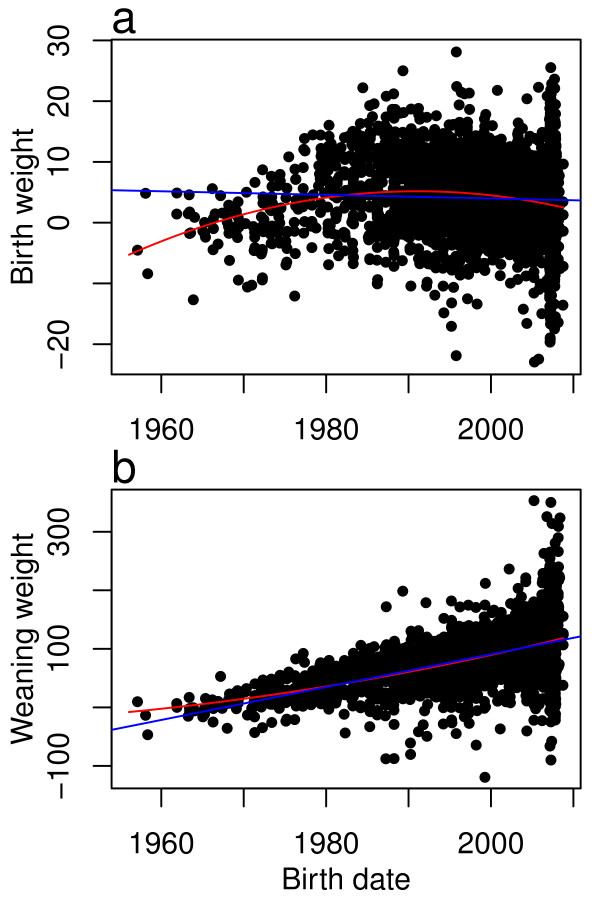
**Deregressed estimated breeding values for birth and weaning weight plotted against birth date.** Deregressed estimated breeding values plotted against birth date for 3,570 Angus animals. The blue lines represent fitted linear and red lines represent fitted quadratic regressions. **a**. Deregressed birth weight EBV, and **b**. Deregressed weaning weight EBV.

Deregressed estimated breeding values (EBVs) [23] for 16 production traits (see Supplementary Information for definitions and acronyms) were regressed on birth date (measured as a continuous variable with month and day converted to a decimal fraction of a year) for 3,570 registered Angus animals (Additional file 1: Table S1, Figure 1 and Additional file 1: Figures S3-S16). For traits that can easily be appraised and for which expected progeny differences (EPDs, an EPD is one half of the EBV) were implemented earlier in the development of the breed (e.g., growth and stature), selection has significantly changed the breed additive genetic mean over time. For traits for which increased production has consistently been sought by producers, such as weaning weight, yearling weight, and milk, additive genetic means have increased linearly (Additional file 1: Table S1; Figure 1b, Additional file 1: Figure S4, and S10). However, additive genetic means for birth weight, yearling height, mature weight, and mature height increased until the mid-1980s when breeders recognized the detrimental effects of large birth weights on calving difficulty and large mature size on cow maintenance requirements and fertility, and these traits were subsequently selected to decrease (Figures 1a, Additional file 1: Figures S5, S11, and S12). For these traits, the quadratic regression models have a much smaller Akaike Information Criterion (AIC), larger adjusted *R*^*2*^ values, and smaller *p*-values (Additional file 1: Table S1). Traits with recently developed EPDs, such as docility and heifer pregnancy rate, show little change in additive genetic mean over time (Additional file 1: Figures S7 and S8). Docility and heifer pregnancy rate had among the smallest *R*^*2*^ values of all the fitted linear and quadratic regression models. Additive genetic means for growth traits (WW, YW, and CW) and for the incidence of unassisted births (CED and CEM) have increased annually. Weaning weight has increased, on average, by 2.81 pounds per year and the rate of unassisted births (CED) has increased by 0.56% per year—remarkable achievements by Angus breeders considering the 50-year span of these data.

### Birth Date Selection Mapping

**Figure 2:**
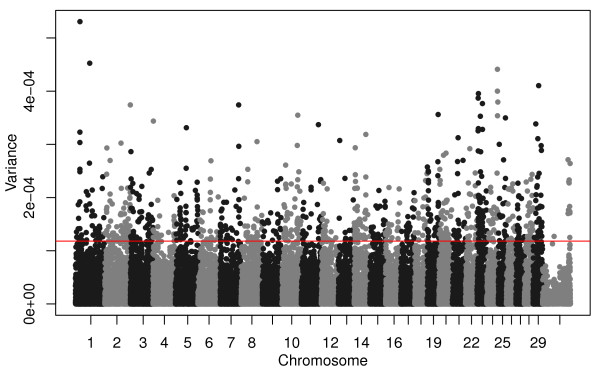
**Manhattan plot of additive genetic variances explained by each SNP estimated from the GBLUP analysis of birth date.** For each SNP 2*p*_*i*_(1-*p*_*i*_)*α*_*i*_^2^is plotted where *p*_*i*_ is allele frequency and *α*_*i*_ is the ASE for birth date for the *i*^*th*^ SNP. Red line corresponds to the top 948 SNPs.

We applied our Birth Date Selection Mapping method to this data set using three mixed models. We first estimated allele substitution effects (ASEs) for birth date for 45,073 SNPs using genomic best linear unbiased prediction (GBLUP) [21,24,25] applied to 3,570 registered Angus cattle, but we do not report results for the 256 SNPs that map to unassigned contigs in the UMD3.1 reference assembly [26]. The GBLUP analysis simultaneously fits all SNPs as random effects and does not estimate *p*-values for tests of significance of individual SNPs. Rather, ASEs were converted to estimates of additive genetic variance associated with each SNP and plotted (Figure 2).

**Figure 3:**
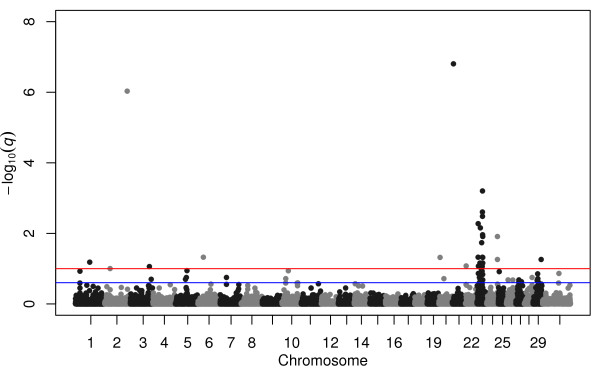
**Manhattan plot of –log**_**10**_**(q-values) for SNP effects on birth date estimated in the EMMAX analysis.** Each *q*-value is the expected proportion of false positives among all SNP effects that are at least as extreme as that observed for the current SNP. Red line corresponds to a FDR of 0.10, and the blue line corresponds to a FDR of 0.25.

We next used EMMAX [20] to individually estimate SNP ASEs as fixed effects and *q*-values representing the expected proportion of false positives among all SNP effects as extreme as observed for the current SNP [27]. Compared to the Poisson regression of allele counts on birth date (Additional file 1: Figure S2), the significance values were not inflated for this analysis (Additional file 1: Figure S17), demonstrating this analysis appropriately models kinship relationships. Additional file 1: Figure S17 further demonstrates that we have sufficient power to identify significant associations. This approach is quite conservative and indicates that strong selection has caused large changes in allele frequency at only a small number of loci on chromosomes 1, 2, 3, 6, 20, 21, 22, 23, 24, and 29 (Figure 3). The two peaks on chromosome 23 contain the major histocompatibility complex (MHC) and numerous olfactory receptors.

**Figure 4:**
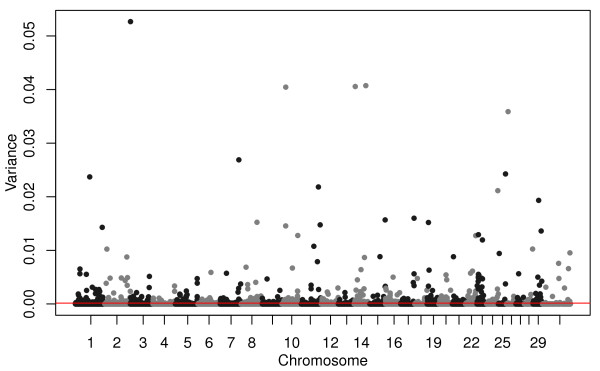
**Manhattan plot of additive genetic variances explained by each SNP estimated from the BayesC*π *analysis of birth date.** Red line corresponds to the top 948 SNPs.

Finally, we used GenSel [28] to fit a non-linear BayesCπ model [29] in which the parameter π estimates the proportion of SNPs that are not associated with the dependent variable. We estimated π to be 0.979, and thus 2.11% (948) of the SNPs were estimated to be predictive of birth date and therefore putatively exposed to strong selection. BayesCπ employs a MCMC approach in which 1-π of the SNPs are sampled for inclusion in the model in each chain and the jointly estimated SNP ASEs are finally shrunk according to the proportion of times each SNP is retained in the selected model. Thus, SNPs that are rarely retained in the model have their ASEs strongly shrunk towards zero. This analysis found strongly selected loci on all chromosomes (Figure 4).

The SNP ASEs estimated with GBLUP and EMMAX had a Spearman correlation of 0.92 and a Pearson correlation of 0.78. The difference in ASE magnitude identified by the Pearson correlation essentially reflects the difference in estimates achieved due to fitting SNPs as uncorrelated random or fixed effects. The SNP ASEs estimated by GBLUP and BayesCπ had a Spearman correlation of 0.85, but had a Pearson correlation of only 0.56 due to the strong shrinkage of large effect SNPs in GBLUP (due to the assumption that all SNPs are drawn from a distribution with a common variance) and the strong shrinkage of small effect SNPs in BayesCπ. The EMMAX and BayesCπ ASEs had a Spearman correlation of 0.83 and Pearson correlation of 0.46. In the GBLUP and EMMAX analyses, the multilocus genotypes explained 0.534 and 0.531 of the variance in birth date (i.e., the “heritability” of birth date), respectively, as estimated using restricted maximum likelihood (REML) estimation of variance components. In the BayesCπ analysis, genotypes explained 0.717 of the variance in birth date.

### Effective population size and drift

**Figure 5:**
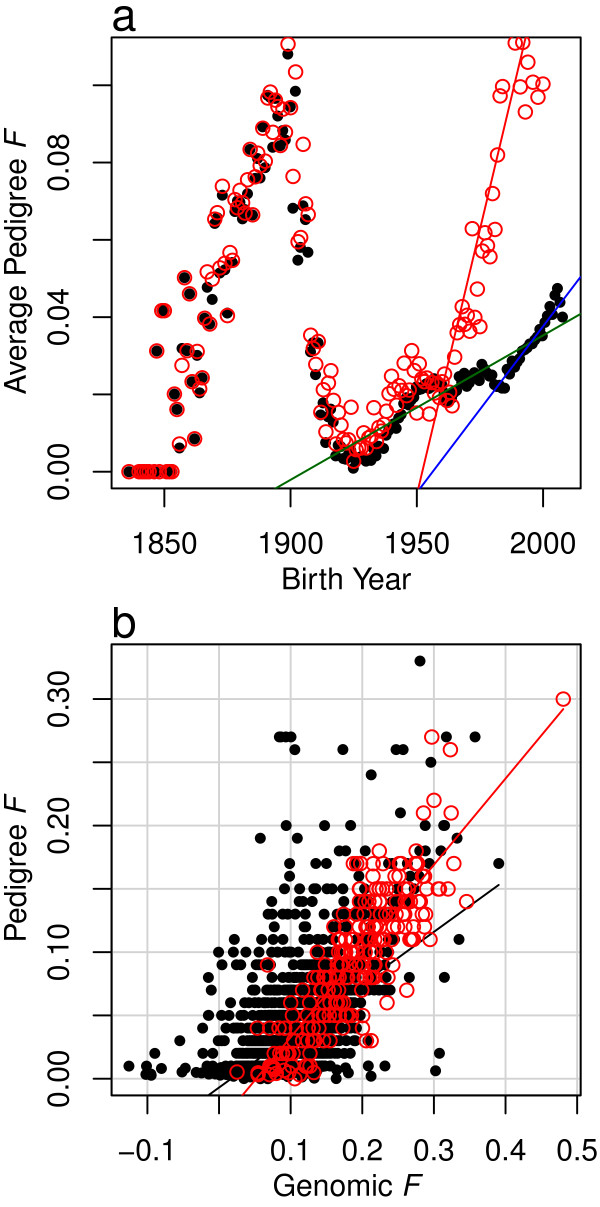
**Analysis of inbreeding coefficients.** a. Plot of average pedigree *F* by birth date for 91,001 animals in the pedigree of the 3,570 genotyped animals. Averages for the Wye herd animals and their ancestors are in red; averages for the remaining North American Angus and their ancestors are in black. The red line represents the regression of pedigree *F* on birth date for Wye pedigree animals born after 1950. The green line is the regression of pedigree *F* on birth date for animals in the North American pedigree born after 1930. The blue line is the regression of pedigree *F* on birth date for animals in the North American pedigree born after 1980. See Table 1 for regression parameter estimates. b. Plot of pedigree against genomic *F* coefficients. Wye herd animals are plotted in red; all other North American animals are plotted in black. The red line represents the regression of pedigree *F* on genomic *F* for Wye herd animals and the black line is for the remaining North American animals. See Table 2 for regression parameter estimates.

To ascertain whether drift is a significant force influencing allele frequency changes within the artificially selected US Angus breed, we estimated the inbreeding effective population size, under the neutral model, and modelled the effects of drift on neutral loci. Using a pedigree of up to 63 generations and which comprised 91,001 Angus animals including the 3,570 genotyped animals and all known ancestors, we estimated the generation interval for US Angus cattle to be 4.99 years, which was the average age of animals born between 1941 and 1990 at the birth of their male and female registered progeny. From this pedigree, we also estimated inbreeding coefficients (denoted as *F*) for all animals from which we estimated effective population size from the regression of *F* on generation number. From a principal component analysis of the SNP genotypes, we identified two distinct subgroups within our sample. In Additional file 1: Figure S18, we identified the Wye Angus herd [30] which was formed from an importation of bulls from the British Isles and then closed to new germplasm in 1958 as a group that was distinct from the remaining US registered Angus cattle. The inbreeding effective population size for the Wye herd was estimated to be 36.41 ± 0.03, whereas the estimate for the remaining North American Angus was 267.59 ± 0.02 using animals born after 1930 and 116.15 ± 0.04 using animals born after 1980 (Figure 5a and Table 1). For each of the 44,817 SNPs, we constructed a test (see Methods) to determine whether the observed change in allele frequency could be explained by drift or alternatively must be due to selection. From this analysis, we found that the observed allele frequency changes exceeded the likely changes due to drift for 84.60% of the 44,817 SNPs.

**Table 1:** Estimates of inbreeding effective population size for registered Angus cattle

**Data set**	**Intercept**	**ΔF/generation**	***N***_***e***_
Wye pedigree, pedigree *F*	−0.0059	0.0137 ± 0.0005	36.4184 ± 0.0338
North American pedigree born after 1930, pedigree *F*	0.0072	0.0019 ± 3.5115e-05	267.5948 ± 0.0188
North American pedigree born after 1980, pedigree *F*	−0.0269	0.0043 ± 0.0002	116.1495 ± 0.0353
North American pedigree born after 1980, genomic *F*	0.0529	0.0052 ± 0.0005	94.1815 ± 0.0955

Birth year generations were calculated by subtracting 1950 from each animal’s birth year and dividing by the generation interval of 5 years. Birth year generation = (birth year – 1950)/5.

We also compared genomic with pedigree estimates of *F*. The realized genomic *F* have a larger variance (s^2^ = 0.0023) than do the pedigree *F* (s^2^ = 0.0014), and the two measures of *F* had a Pearson correlation of 0.65 (Figure 5b), which is consistent with the underestimation of pedigree *F* due to the assumption that *F* is zero for all individuals in the base generation, pedigree errors, and incomplete pedigree information. We regressed pedigree *F* on genomic *F*, and found the slope of the regression to be 0.49 ± 0.01. Separate regressions for the Wye and North American Angus revealed pedigree and genomic *F* coefficients to be more similar for the Wye herd than for the remaining North American Angus cattle (see Figure 5b and Table 2). When genomic *F* was regressed on pedigree *F*, the slope of the regression was 0.85 ± 0.02 with an adjusted *R*^2^ of 0.42.

**Table 2:** **Regression of pedigree *F *on genomic *F***

**Sample**	**Adjusted *R***^***2***^	**Model *p*-value**	**Parameter**	**Estimate**	**T-value**	***p*-value**
All	0.4178	< 2.2e-16	Int	−0.0145 ± 0.0012	−12.46	< 2e-16
			Slope	0.4912 ± 0.0097	50.61	< 2e-16
Wye	0.7077	< 2.2e-16	Int	−0.0354 ± 0.0060	−5.93	1.18e-8
			Slope	0.6813 ± 0.0296	23.00	< 2e-16
North American	0.2829	< 2.2e-16	Int	−0.0070 ± 0.0013	−5.53	3.48e-8
			Slope	0.4100 ± 0.0113	36.36	< 2e-16

Because estimates of genomic *F* coefficients are based on fewer assumptions than are pedigree estimates (which require neutrality), we also estimated *N*_*e*_ using the genomic *F* coefficients for North American Angus animals born after 1980. This resulted in an *N*_*e*_ of 94.18 ± 0.10 (Table 1). Using this *N*_*e*_ in our drift test, we estimated that allele frequency changes exceeded those likely due to drift for 82.41% of the 44,817 SNPs.

### Applying Birth Date Selection Mapping to smaller sample sizes

We appreciate that not all species will have the large samples that are available for agriculturally important species for Birth Date Selection Mapping. To assess the effects of sample size and birth date range, we created four subsamples from our data set that were analysed using EMMAX. The first subsample contained 1,237 animals from pedigree generations 58, 59, and 60 (mean birth date 2004.84 ± 3.03; range 1993.16 to 2008.66). The second contained 60 animals, consisting of 20 animals randomly sampled from each of pedigree generations 58, 59, and 60 (mean birth date 2005.16 ± 2.20; range 1999.14 to 2008.01). The third consisted of 1,237 animals randomly sampled from the entire data set (mean birth date 1999.10 ± 9.00; range 1955.99 to 2008.64). The final sample included 60 animals randomly sampled from the entire data set (mean birth date 1998.77 ± 8.56; range 1974.34 to 2008.10). Additional file 1: Figures S19 and S20 show that only the third data set had sufficient power to identify loci under selection with genome-wide significance. Comparing Additional file 1: Figures S20A and S20D suggests that increasing the time period over which individuals are sampled improves power more than does increasing sample sizes using contemporary individuals. Selection would likely have to be extremely strong and focussed on monogenic traits to identify selected loci using small samples of contemporaries (Additional file 1: Figures S19B and S20B).

### Connecting selected phenotype to selected genotype

**Figure 6:**
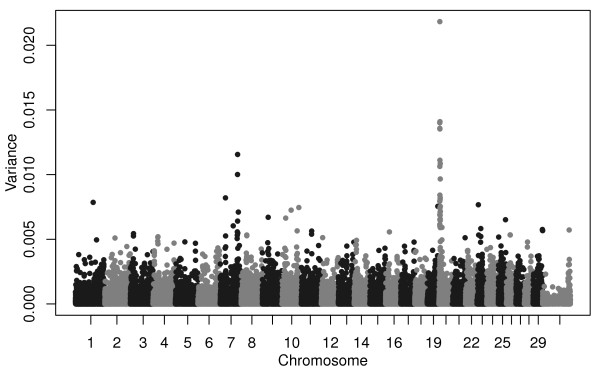
**Manhattan plot of additive genetic variances explained by each SNP estimated from the GBLUP analysis of deregressed weaning weight EBVs.** For each SNP 2*p*_*i*_(1-*p*_*i*_)*α*_*i*_^2^is plotted where *p*_*i*_ is allele frequency and *α*_*i*_ is the ASE for weaning weight for the *i*^*th*^ SNP.

We analysed deregressed EBVs in a weighted analysis [23] for 16 production traits (Supplementary Information) using data provided by the American Angus Association (AAA) under an animal model that incorporated a genomic relationship matrix and from which we estimated the proportion of additive genetic variance explained by the SNP markers (Table 3). With the exception of two QTLs on chromosomes 7 and 20, most genes influencing variation in growth traits in Angus cattle are of small effect (Figures 6, Additional file 1: Figures S21-S35). The most likely location of the pleiotropic QTL on chromosome 7 was found to be at 93.22 Mbp in the GBLUP analyses, and the SNP at this location, *rs110059753*, was among the most strongly associated of all SNPs with CED, BW, WW, YW, HP, CEM, MILK, MW, MH, CW, MARB, RE, and FAT (Figures 6, Additional file 1: Figures S21, S22, S23, S27, S28, S29, S30, S31, S32, S33, S34, S35). However, the strongest selection signal found on this chromosome was found at 99.02 Mbp (Figure 2) by GBLUP, at 100.02 Mbp by BayesCπ (Figure 4), and a small, but not significant, birth date selection signal was found at 99.02 Mbp in the EMMAX results (Figure 3). The birth date effect for *rs110059753* was ranked 11,224 out of the 44,817 SNP effects (75^th^ ASE percentile). The most likely location of the pleiotropic QTL on chromosome 20 was estimated to be at the position of SNP *rs43711332* at 4.62 Mbp (affecting CED, BW, WW, YW, YH, CEM, MW, MH, and CW; Figures 6, Additional file 1: Figures S21, S22, S23, S24, S28, S30, S31, and S32). Selection signals were detected at 5.1 Mbp in the GBLUP and at 5.9 Mbp in the EMMAX analyses of birth date. The birth date effect for SNP *rs43711332* was ranked 5,168 out of the 44,817 SNP effects (88^th^ ASE percentile).

**Table 3:** **Summary statistics for deregressed estimated breeding values (EBVs) and accuracies (r**^**2**^**) produced by the American Angus Association for the 3,570 genotyped animals**

**Trait**^**1**^	**Units**	**Heritability**^**2**^	**N**^**4**^	**Mean EBV ± SD**^**5**^	**Mean Accuracy ± SD**	**C**_**max**_^**6**^	**C**^**7**^	**V**_**genetic**_^**8**^
Birth Weight	lb	0.42	3241	4.03 ± 5.95	0.78 ± 0.24	0.7962	0.7703	23.42
Weaning Weight	lb	0.20	3229	86.69 ± 45.98	0.68 ± 0.32	0.8221	0.7038	690.86
Maternal Milk	lb	0.14	2067	33.79 ± 30.01	0.70 ± 0.27	0.8619	0.7086	373.15
Yearling Weight	lb	0.49	2776	154.03 ± 78.15	0.69 ± 0.29	0.8268	0.7804	1961.63
Yearling Height	in	0.45	2250	0.74 ± 1.22	0.70 ± 0.25	0.7962	0.7962	0.6165
Carcass Weight	lb	0.40	2457	30.93 ± 84.08	0.41 ± 028	0.9141	0.6274	1438.86
Marbling	units	0.45	3237	0.64 ± 1.14	0.43 ± 0.25	0.9127	0.9127	0.3542
Ribeye Muscle Area	in^2^	0.51	3269	0.16 ± 1.04	0.47 ± 0.23	0.9141	0.9141	0.3775
Fat Thickness	in	0.34	3189	0.027 ± 0.162	0.40 ± 0.23	0.9141	0.9141	0.0072
Mature Weight	lb	0.55	1321	67.28 ± 135.26	0.64 ± 0.25	0.8485	0.5586	5818.80
Mature Height	in	0.82	1291	1.08 ± 2.25	0.64 ± 0.25	0.8429	0.5602	1.504
Scrotal Circumference	in	0.43	2479	0.55 ± 1.83	0.69 ± 0.25	0.8176	0.6977	1.641
Calving Ease Direct	%	0.18	3217	8.30 ± 19.77	0.62 ± 0.26	0.8681	0.7055	154.70
Calving Ease Maternal	%	0.12	1966	12.14 ± 23.77	0.59 ± 0.27	0.9026	0.4211	146.00
Docility	%	0.37	698	15.52 ± 21.44	0.48 ± 0.27	0.9267	0.3430	126.94
Heifer Pregnancy	%	0.13	1366	15.81 ± 47.64	0.50 ± 0.27	0.9049	0.7117	711.45
Birth Date	yr	0.53^3^	3570	1998.93 ± 8.98	1.00 ± 0.00	N/A	N/A	25.83

^1^See Supplementary Information for trait definitions.

^2^Narrow sense heritability used by the American Angus Association to compute estimates of additive genetic merit.

^3^Estimated from genomic BLUP analysis.

^4^Number of breeding values that could successfully be deregressed or birth dates.

^5^Deregressed estimated breeding values or birth dates.

^6^Largest possible value of C imposed by the constraint (1+F_i_)/r_i_^2^>C×G_ii_, which ensures that weights for all animals’ deregressed EBV are strictly positive. F_i_ is the pedigree inbreeding coefficient, r_i_^2^ is the accuracy of the deregressed breeding value, and G_ii_ is the diagonal of the genomic relationship coefficient matrix for the i^th^ animal. See [23] for further explanation.

^7^Proportion of additive genetic variation explained by 45,073 SNPs computed as V_markers_/V_genetic_. See [23] for further explanation.

^8^Estimated additive genetic variance from the analysis of deregressed breeding values or birthdates.

To assess the identity of the trait or combination of traits that have historically been under selection in Angus cattle and that produced the molecular signals of selection, we individually regressed the SNP ASEs for birth date on standardized SNP ASEs (see Methods) for all 16 production traits for which the AAA routinely produces EPDs (Additional file 1: Table S2) using generalized least squares accounting for the pair-wise LD among SNPs. We fit this model for the 948 SNPs (935 SNPs after LD pruning) with the largest birth date variances corresponding to the 1- $\hat{\pi }$= 0.0211 proportion of SNPs detected to be under strong selection in the BayesCπ analysis of birth date. Growth traits (WW, YW, and CWT), milk, marbling and calving ease (CED and CEM) had the largest adjusted coefficients of determination and relative selection intensities (Additional file 1: Table S2). However, the models for birth weight, docility, and yearling height were not significant (Bonferroni corrected α = 0.003125 = 0.05/16). A multiple regression with all 16 traits fit jointly produced an adjusted *R*^*2*^ of 0.6625, which is only slightly (0.0457) larger than the *R*^*2*^ for the weaning weight model. Individual terms from the multiple regression are not reported due to multicollinearity between several traits.

Finally, to elucidate the biological processes associated with the genes located in the genomic regions detected to be under selection, we analysed the gene ontology term enrichment for the annotated genes within these regions (See Additional file 2 and Additional file 3). From the GBLUP results, we queried 2,074 genes within 100 Kbp of the top ranked 948 SNPs for their birth date ASEs, and from the BayesCπ results, we queried 1,996 genes within 100 Kbp of the top 948 SNPs. There were 1,059 genes shared between the two lists, and this list of genes was also queried using the DAVID Bioinformatics tools. Various biological processes appear to be under selection based on the intersection of the GBLUP and GenSel results—notably cellular metabolic process, biosynthetic process, translation, protein folding, regionalization, ectoderm development, leukocyte mediated immunity, and striated muscle cell proliferation (Additional file 4 contains the complete list). The intersection of the GBLUP and GenSel results also found olfactory transduction, antigen processing and presentation, and the adipocytokine signalling pathway to be enriched KEGG pathways. In addition to these terms, cell proliferation, spermatogenesis, and organ growth were enriched gene ontology terms from the GBLUP results (Additional file 2 contains the complete list). Developmental process, anatomical structure development, cellular response to stress, response to oxidative stress, positive regulation of lymphocyte activation, and limb morphogenesis were additional enriched gene ontology terms from the GenSel results (Additional file 3 contains the complete list).

## Discussion

Artificial selection has increased the weights at which cattle are marketed either at weaning or yearling ages (Figures 1, Additional file 1: Figures S4, and S13) while simultaneously decreasing the incidence of assisted births (Additional file 1: Figures S3 and S9), and the trends observed in our data set are very similar to those reported for the entire Angus breed [31]. Larger birth weights and yearling heights are both strongly associated with increased calving difficulty and genetic trend increased both traits until about the mid-1980s, after which both began to decrease (Figures 1 and Additional file 1: Figures S5). Breeders did not directly select to increase birth weight, but it increased as a correlated response to selection for increased weaning and yearling weights. Some breeders selected for increased yearling height to produce Angus cattle more comparable in frame size to the Continental European breeds, which were imported into the US during the 1970s [32]. However, once breeders appreciated the undesirable correlated response in calving ease, selection was practised to increase weaning and yearling weights while maintaining birth weight and yearling height constant.

Using EMMAX, only eleven loci were found to be significantly associated with birth date and thus under strong selection, but all loci simultaneously explained 53% of the variation in birth date. On the other hand, BayesCπ estimated that 2.11% of the SNPs were strongly associated with birth date, but all SNPs explained 72% of the variance in birth date. The difference in the heritability estimates between the GBLUP or EMMAX analyses compared to the BayesCπ analysis reflects the different model assumptions underlying these analyses. Whereas GBLUP and EMMAX assume the infinitesimal model under which all SNP ASEs are drawn from a distribution with constant variance [33,34], BayesCπ begins with a distribution with constant variance but shrinks the variance for small effect SNPs that are rarely fit in the model. As a consequence, GBLUP and EMMAX regress all SNP ASEs equally towards the mean of zero, while BayesCπ more aggressively regresses small effect SNPs and less aggressively regresses large effect SNPs, leading to a better model fit—as was found here—when there are loci under very strong selection. In the absence of selection, genotype frequency should be independent of time provided that the effects of drift are negligible and the heritability estimates should be close to zero. However, this was not the case for US registered Angus cattle and we conclude that a significant number of loci are rapidly responding to selection (our results suggest 2.11%) and that most of the genome (82.4% from the drift analysis) is responding more slowly to selection. Furthermore, in our nonlinear BayesCπ model, 72% of the variation in birth date could be explained by simultaneously using all SNP genotypes, suggesting that there are loci under very strong selection (large effect loci) that are not appropriately fit by the infinitesimal model.

The SNP ASEs for the 16 analysed traits indicate that, with the exception of the two large effect QTLs on BTA 7 and 20, the vast majority of QTLs underlying quantitative traits in beef cattle are of small effect. Of considerable interest, neither of these QTLs was found to be under very strong selection and this seems to be because of their large antagonistic pleiotropic effects on growth and calving difficulty. We postulate that when multiple traits are simultaneously selected, the genetic architecture of the population defined by pleiotropy and the chromosomal organization of QTL alleles (phase effects) constrains both the phenotypic and genotypic response to selection.

For selection to be effective, the selection intensity and effective population size must be sufficiently large to overcome the effects of genetic drift. We demonstrate that US registered Angus cattle have a sufficiently large effective population size to enable successful artificial selection, but more importantly, that large intergenerational changes in allele frequency are unlikely to occur due to drift alone. Furthermore, we found a considerable disparity between pedigree and genomic estimates of inbreeding coefficients. While others have argued that genomic relationship matrices should be adjusted to more closely resemble pedigree relationship matrices [35], we assert that genomic relationship matrices provide a more accurate representation of the realized relationships among individuals that result from the Mendelian sampling of parental gametes and selection. The use of genomic relationship matrices in place of pedigree relationship matrices avoids the assumption of neutrality of loci both in the estimation of inbreeding coefficients and for the mean value of gametes inherited by progeny—both of which are assumed for the computation of the numerator relationship matrix [36]. The disagreement between genomic and pedigree estimates of *F* coefficients is likely to be due to the assumption that base animals are not inbred, errors in the pedigrees, and missing pedigree information likely due to the large-scale importation of Canadian Angus cattle in the 1940s and 1950s that were not carriers of dwarfism alleles, which had been driven to high frequency due to selection at the time [37]. This is supported by the closer agreement between pedigree and genomic *F* coefficients for the Wye herd that was largely derived from British stock with more complete pedigree records than the remaining US registered Angus cattle (Table 2 and Figure 5b).

We attempted to identify the relative selection intensities placed on each selected trait via the imprints that multi-trait selection had left on the Angus genome. Although this analysis assumed no change in relative selection intensities in time, an assumption that is clearly violated in view of the genetic trends in birth weight and yearling height, we were able to confirm that growth traits have historically been the most strongly selected in US registered Angus cattle. Because Angus is considered to be a maternal breed (i.e., motherly, used as dams in commercial beef production), it is logical that loci that influence calving ease, growth to weaning, and milking ability should have been found to be under selection. Angus breeders have successfully selected to increase calving ease and body weight by selecting for body shapes that allow a calf’s easy passage through the dam’s pelvis. This is supported by the finding of an enrichment of gene ontology terms related to limb morphogenesis and anatomical structure development within regions of the genome detected as responding to selection. It has previously been shown that calving ease is negatively correlated with several body measures, such as head circumference, head width, hip width, hip height, chest girth, and cannon bone circumference [38-40]. Likely due to the Certified Angus Beef’s emphasis on quality grade [41], Angus breeders have more recently selected to increase marbling. Conversely, traits such as fat thickness, docility, and heifer pregnancy rate have not been as intensely selected as growth traits, due to the differing breeding objectives of beef producers, genetic antagonisms constraining selection response, and the historic difficulty in collecting field data to allow the development of EPDs for these traits.

There is also evidence that natural selection has occurred in this population. The gene ontology enrichment results indicate that genes affecting immune response, such as the MHC, *NOD2*, *C3*, and *DBH*, have strongly responded to selection (Additional file 2, Additional file 3, and Additional file 4), presumably due to the exposure of Angus cattle to novel pathogens following their introduction into the US in 1873 [42] and the continuous co-evolutionary “arms race” between bovine and pathogen genomes [43,44]. The Bovine HapMap Consortium [12] found that the MHC had some of the lowest F_st_ values in the entire genome when compared between breeds. Our analyses have identified the MHC as being under strong selection. Taken together, these results suggest that the MHC or certain of the numerous olfactory receptors which occupy the same region on chromosome 23 are under strong convergent selection.

Furthermore, natural selection may also be attempting to buffer the cellular environment against the deleterious effects of inbreeding. We found that spermatogenesis was an enriched ontology term describing the function of genes within the strongly selected regions of the genome (Additional file 3). Seminal plasma proteins have been associated with bull fertility [45], and selection may be increasing fertility to counter act the inbreeding depression of reproduction (alternatively, the use of AI may be selecting for increased fertility). Genes involved in response to oxidative stress were also identified; response to oxidative stress has been tied to mitigating inbreeding depression [46]. We also inferred that at least 6 heat shock proteins are under selection in Angus and that protein folding was an enriched biological process (Additional file 4). It has been hypothesized that heat shock proteins assist the organism to cope with protein instability and misfolding caused by homozygous nonsynonymous mutations that are elevated in frequency by inbreeding [46-50].

One of the greatest difficulties encountered in identifying genomic signatures of selection is in distinguishing changes that have occurred due to demographic as opposed to selective forces [9]. Our Birth Date Selection Mapping approach utilizing mixed models specifically accounts for pedigree relationships and explicitly deconvolutes their confounding effects on time-dependent allele frequency changes, which are due to the fact that not all pedigrees are sampled equally deeply in terms of the numbers of genotyped individuals. Rather than fit generations as the dependent variable [51], which are poorly estimated when pedigrees are incomplete, fitting birth date allows unknown or complex pedigrees with overlapping generations to be analysed. Furthermore, the genomic relationship matrix accounts for pedigree relationships between samples, allowing closely related samples to be analysed. However, one of the limitations of Birth Date Selection Mapping is the requirement of a temporally stratified sample of genotyped individuals. The results for the analyses of the reduced data subsets suggest that sampling over extended time periods or large sample sizes—but not necessarily both—will be necessary to identify strongly selected loci. This will currently limit the utility of the approach in human populations due to a lack of preserved DNA samples across multiple generations. However, this limitation may be alleviated as it becomes more practical to extract quality DNA from formalin-fixed, paraffin-embedded tissue section samples and ancient remains. Nevertheless, Birth Date Selection Mapping is clearly most easily applied to organisms with temporally stratified DNA samples and genome-wide genotypes.

Using the estimated birth date ASEs as informative priors in the development of genomic selection programs [52] is another interesting application of our method. Loci with small birth date ASEs are either of small effect on the selection objective or represent genes of large effect that have undesirable pleiotropic effects (or closely linked loci with antagonistic phase relationships). Loci that have large birth date ASEs must have large effects on the selection objective that are less constrained by antagonistic pleiotropic effects allowing them to more rapidly respond to selection.

## Conclusions

When temporally stratified DNA samples are available, Birth Date Selection Mapping is an effective method for the identification of strongly selected loci. We demonstrate that selection on polygenic traits leaves detectable signatures of selection throughout the genome at which small changes in allele frequencies per generation have occurred. If genes with large antagonistic pleiotropic effects exist, they respond to multi-trait selection as if they were of small effect on the breeding objective as predicted by quantitative genetic theory. By relating the detected signatures of selection to phenotype, we infer that artificial selection in US registered Angus cattle has historically focussed primarily on growth and maternal traits including calving ease, weaning weight, and milking ability. This result was directly confirmed by the response to selection that has occurred in these traits that we directly estimated from EPDs provided by the AAA. Finally, our results suggest that natural selection has also acted in this domesticated population to increase immunity and possibly to buffer the organism against the effects of inbreeding depression.

## Methods

### DNA extraction and SNP genotyping

Cryopreserved semen was obtained from semen distributors, the National Animal Germplasm Program, and individual Angus breeders including the University of Maryland Foundation which owns the Wye herd. DNA was extracted using a proteinase K digestion, Phenol: Chloroform alcohol extraction, and ethanol precipitation [53]. Single nucleotide polymorphisms were assayed using the Illumina BovineSNP50 BeadChip [54] and genotyped using the Illumina GenomeStudio software. Genotypes were filtered using a SNP call rate threshold of 90%, animal call rate threshold of 95%, and minor allele frequency threshold of 0.01. Autosomal and pseudoautosomal SNPs that had a Hardy-Weinberg Chi-square statistic with 1 degree of freedom greater than 300 were also filtered—primarily to remove polymorphisms detected in copy number variants rather than remove loci that were under selection [25]. Filtered data were processed through FastPHASE version 1.4.0 [55] to impute the 0.49% of missing genotypes. Parameter values were set at T=10, K=20, with -eo flags set. The resulting dataset consisted of genotypes for 45,073 SNPs scored in 3,570 animals with no missing values.

### Response to selection

Expected progeny differences for 16 production traits along with their accuracies were provided by the AAA for 103,816 animals including the 3,570 genotyped animals and all identified ancestors in their pedigrees. These values were doubled to obtain estimated breeding values that were deregressed for the 3,570 animals as previously described [23]. The deregression of estimated breeding values removes parent average information and converts the information available on the individual back to the scale of the underlying phenotype—that is, it removes the “shrinkage” that was applied to convert phenotypes into estimated breeding values. In the statistical package R [56], trait breeding values were plotted against birth date. Linear and quadratic regressions were fit for each trait.

### Principal component analysis of Angus genotypes

We used the *smartpca* program, part of EIGENSOFT [57], for principal component analysis of the Angus genotypes. We plotted principal component 1 by principal component 2 to visualize the largest elements of population substructure. Figure S18 revealed that the primary substructure detected in the population was the largest families—the linearly related members of the Wye herd and the ancestors and sons of N Bar Emulation EXT, a popular bull within the breed that generated numerous sons also used in artificial insemination.

### Estimation of effective population size

Pedigree inbreeding coefficients (*F*) were estimated as a by-product of using the rapid algorithm for producing the inverse of a numerator relationship matrix [36]. Genomic *F* were estimated by subtracting 1 from the diagonals of the genomic relationship matrix, which was estimated according to [24] with base generation allele frequencies at each SNP estimated using the 59 animals born between 1955 and 1974, excluding all animals from the Wye herd.

The inbreeding effective population size *N*_*e*_ was estimated from the regression of inbreeding coefficients on pedigree generation number using individual animal data. This requires inverting the relationship *ΔF* = 1/2*N*_*e*_, in which *ΔF* is the increase in mean inbreeding coefficient between adjacent generations [58] and is estimated as the slope of the regression across all generations if *N*_*e*_ is assumed constant in time. A Taylor series expansion leads to an estimate of the standard error of *N*_*e*_ as *SE*(*N*_*e*_) ≈ 2*N*_*e*_*SE*(*ΔF*), in which *SE*(Δ*F*) is the standard error of the estimated slope of the regression. Because the depth of available pedigree information varied substantially for the 3,570 sampled Angus animals (animals within the pedigree that were assigned to generation 0 varied in birth year from 1838 to 1954) we considered the estimates of pedigree generation to be unreliable from the perspective of estimating *N*_*e*_. Accordingly, we estimated generation number for each of the 3,570 genotyped animals by subtracting 1950 from their birth year and dividing by the generation interval of 5 years. Because of the closed nature of the Wye herd and complete pedigree information back to foundation animals, we fit separate models for the Wye and remaining North American Angus animals. For the North American Angus subset, we fit two models using generation number estimated from birth year for animals born after 1930 and for animals born after 1980 where there appeared to be an inflection in the rate of increase in inbreeding. This corresponds to the point in time when the increased use of artificial insemination became significant within the breed.

For each of the 44,817 SNPs, we directly estimated the change in allele frequency that occurred between the 460 individuals assigned to pedigree generation 58 and the 450 individuals assigned to pedigree generation 59 using PLINK [59,60]. These pedigree generations were chosen because they had the largest sample sizes, represent the individuals with the deepest pedigrees for which their generation assignment would not be significantly influenced by missing pedigree information, and were among the most recent of the generations suggesting they were likely to represent all of the families present within the sample. Furthermore, the sample sizes for these generations were sufficiently large to obtain precise estimates of allele frequencies. We compared the observed allele frequency changes between generations 58 and 59 to the boundaries of the 99.999999% (−log_10_(*p*-value) = 8) confidence interval for the change in allele frequency due to drift (estimated under the assumption of normality assuming a mean of 0 and the drift variance for the *i*^*th*^ SNP to be *p*_*i*_(1-*p*_*i*_)/2*N*_*e*_, for *p*_*i*_ the frequency of the *A*_*i*_ allele in generation 58 and *N*_*e*_ = 116.15 [58]). For SNPs on the X chromosome, the drift variance for the *i*^*th*^ SNP was *p*_*i*_(1-*p*_*i*_)/1.5*N*_*e*_. Loci for which the allele frequency change exceeded the boundaries of the confidence interval were concluded to be changing in frequency due to selection rather than drift.

### GBLUP of phenotypic traits

In a weighted analysis using deregressed EBVs as previously described [23], GBLUP [24] was used to estimate ASEs for 16 different traits using 45,073 SNPs genotyped in 3,570 animals. Allele substitution effects were converted to additive genetic variances by squaring the ASE and multiplying by 2*p*_*i*_*q*_*i*_, in which *p*_*i*_ and *q*_*i*_*= 1 - p*_*i*_ are the base generation allele frequencies at the *i*^*th*^ SNP [24]. Base generation allele frequencies at each SNP were estimated using the 59 animals born between 1955 and 1974, excluding all animals from the Wye herd. Results are presented only for the 44,817 SNPs that mapped to autosomes or the X chromosome in the UMD3.1 bovine assembly.

### Signatures of selection analysis

SNPs with the greatest changes in allele frequency over time will have the largest ASEs for birth date. The ASE reflects the amount of response to selection realized by the genomic region tagged by a SNP. Genome-wide associations with birth date were analysed by GBLUP using custom developed software described in [21], by EMMAX [20], and by BayesCπ [29] as implemented in GenSel [28]. A genomic relationship matrix [24], computed as previously described, was incorporated in the GBLUP analysis and a Balding-Nichols matrix [61] was used as the kinship matrix in the EMMAX analysis. Both analyses estimated the amount of variation in birth date explained by multilocus genotypes using REML [20]. Test statistic *p*-values for each SNP produced by EMMAX were adjusted to *q*-values using the method of Benjamini and Hochberg [62] as implemented by the GenABEL package in R [63]. The additive genetic and residual variance components estimated in the GBLUP analysis were used as starting values for variance components in the BayesCπ analysis. The starting value for π was set to 0.9 and GenSel was run for 160,000 iterations, with 1,000 iterations used as burn-in. Manhattan plots were created in R [56], with R source code from [64] which was altered to allow 30 chromosomes on the X-axis and for *q*-values or variances to be plotted on the Y-axis.

From Falconer and MacKay [58], the change in allele frequency resulting from selection is Δ*q* = −*ipqa*/σ_*p*_, where *i* is the selection intensity, *a* is one half the phenotypic difference between homozygote mean phenotypes, σ_*p*_ is the trait variance, and *p* and *q* are allele frequencies. Assuming the dominance deviation is zero, the ASE α is equal to the genotypic value *a*. Thus, we use the ASE as a proxy for *a* which we then scaled as *pq*ASE/σ_*ASE*_ to form the independent variables for each of the 16 production traits which were individually regressed on the birth date ASEs to provide estimates of the relative selection intensity *i* for each trait (the sign is included in the realized estimate). For each trait, σ_*ASE*_ represents the ASE standard deviation in the equation above. Regressions were performed using generalized least squares, with **e** ~ (0, **V**σ) where **V** was the matrix of correlations between alleles at pairs of SNPs estimated using PLINK. When multiple, contiguous SNPs had r = ±1 (i.e., r^2^ = 1), only the first SNP was retained, resulting in the removal of 13 SNPs. The model was fit to the 935 SNPs with the largest birth date ASEs.

### Functional annotation

Due to the extent of LD within the bovine genome [12,65], we identified all genes within 100 Kbp of the 948 most strongly selected SNPs (top 2.11% of 44,817 SNPs, estimated by BayesCπ) identified by the GBLUP and BayesCπ analyses. We used the DAVID bioinformatics resources [66,67] to identify enriched GO terms in the lists of 2,074 (GBLUP) and 1,996 (BayesCπ) genes, and the 1,059 genes in common between the lists. We used annotations from *Bos taurus*, *Homo sapiens*, *Mus musculus*, *Rattus norvegicus*, *Canis lupus*, *Pan troglodytes*, *Macaca mulatta*, *Equus caballus*, *Pongo abelii*, *Sus scrofa*, *Ovis aries*, and *Oryctolagus cuniculus* for GO enrichment analysis.

## Competing interests

The authors declare that they have no competing interests.

## Authors' contributions

JFT and JED created the methodology and designed the experiment. DAV, JED and JFT analyzed data. SDM, MCM, MMR, JWK, and RDS extracted DNA. MMR and SDM prepared samples for genotyping, SDM ran the Illumina assay, and RDS genotyped samples and managed the genotype database. SLN provided pedigree and estimated genetic merit data and SB and BWW provided genotypes on about 900 Angus animals. JED and JFT wrote the manuscript and other authors provided feedback. All authors read and approved the final manuscript.

## Data availability

Genotypes are available to scientists interested in non-commercial research upon signing a Materials Transfer Agreement (MTA).

## Supplementary Material

Additional file 1 **Supplementary Material.** File includes supplementary information, supplementary figures 1 through 35, and supplementary Tables 1 and 2. Click here for file

Additional file 2 **Chart of enriched GO terms in Excel xlsx format.** We included charts for DAVID’s GOTERM_BP_FAT (lower levels of biological process ontology), GOTERM_BP_ALL (all levels of biological process ontology), GOTERM_BP_2 (2nd level of biological process ontology), GOTERM_BP_3 (3rd level of biological process ontology), GOTERM_BP_4 (4th level of biological process ontology), GOTERM_BP_5 (5th level of biological process ontology), GOTERM_CC_FAT (lower levels of cellular component ontology), GOTERM_MF_FAT (lower levels of molecular function ontology), and KEGG_PATHWAY with each as an individual tab in the file. We supplied the DAVID resources with a list of 2,074 genes annotated in the UMD 3.1 assembly. Click here for file

Additional file 3 **Chart of enriched GO terms in Excel xlsx format.** We included charts for DAVID’s GOTERM_BP_FAT, GOTERM_BP_ALL, GOTERM_BP_1, GOTERM_BP_2, GOTERM_BP_3, GOTERM_BP_4, GOTERM_BP_5, GOTERM_CC_FAT, GOTERM_MF_FAT, and KEGG_PATHWAY with each as an individual tab in the file. We supplied the DAVID resources with a list of 1,996 genes annotated in the UMD 3.1 assembly. Click here for file

Additional file 4 **Chart of enriched GO terms in Excel xlsx format.** We included charts for DAVID’s GOTERM_BP_FAT, GOTERM_BP_ALL, GOTERM_BP_1, GOTERM_BP_2, GOTERM_BP_3, GOTERM_BP_4, GOTERM_BP_5, GOTERM_CC_FAT, GOTERM_MF_FAT, and KEGG_PATHWAY with each as an individual tab in the file. We supplied the DAVID resources with a list of 1,059 genes annotated in the UMD 3.1 assembly. Click here for file
