# Supplementary material for: A novel analytical method, Birth Date Selection Mapping, detects response of the Angus (Bos taurus) genome to selection on complex traits
Source: BMC Genomics. 2012 Nov 9;13:606. doi: 10.1186/1471-2164-13-606 (PMC3532096; doi:10.1186/1471-2164-13-606)
Supplement: Additional file 1 — Supplementary Material. File includes supplementary information, supplementary figures 1 through 35, and supplementary Tables 1 and 2. [file 1471-2164-13-606-S1.docx]

# Supplementary Material

# A novel analytical method, Birth Date Selection Mapping, detects response of the Angus (*Bos taurus*) genome to selection on complex traits

### Jared E. Decker^1^, Daniel A. Vasco^1,2^, Stephanie D. McKay^1,3^, Matthew C. McClure^1,4^, Megan M. Rolf^1,5^, JaeWoo Kim^1^, Sally L. Northcutt^6^, Stewart Bauck^7^, Brent W. Woodward^8^, Robert D. Schnabel^1^, Jeremy F. Taylor^1§^

^1^Division of Animal Sciences, University of Missouri, Columbia, MO 65211, USA

^2^Biology Department, Duke University, Durham, NC 27708, USA

^3^Department of Animal Science, University of Vermont, Burlington, VT 05405, USA

^4^Bovine Functional Genomics Laboratory, ARS, USDA, Beltsville, MD 20705, USA

^5^Department of Animal Science, Oklahoma State University, Stillwater, OK 74078, USA

^6^American Angus Association, 3201 Frederick Ave, Saint Joseph, MO 64506, USA

^7^GeneSeek, 4665 Innovation Drive, Suite 120, Lincoln, NE 68521, USA

^8^NextGen, Duluth, GA 30096, USA

^§^Corresponding author

Email addresses:

JED: [deckerje@missouri.edu](mailto:jed42d@mail.missouri.edu)

DAV: [daniel.vasco@duke.edu](mailto:daniel.vasco@duke.edu)

SDM: [stephanie.mckay@uvm.edu](mailto:stephanie.mckay@uvm.edu)

MCM: [Matthew.McClure@ars.usda.gov](mailto:Matthew.McClure@ars.usda.gov)

MMR: [mrolf@okstate.edu](mailto:mrolf@okstate.edu)

JWK: [kijae@missouri.edu](mailto:kijae@missouri.edu)

SLN: [snorthcutt@angus.org](mailto:snorthcutt@angus.org)

SB: [sbauck@neogen.com](mailto:sbauck@neogen.com)

BWW: [bww25@cornell.edu](mailto:bww25@cornell.edu)

RDS: [schnabelr@missouri.edu](mailto:schnabelr@missouri.edu)

JFT: [taylorjerr@missouri.edu](mailto:taylorjerr@missouri.edu)

## Supplementary Information

The following definitions and abbreviations include excerpts from: http://www.angus.org/Nce/Definitions.aspx.

*Expected Progeny Difference (EPD)*. Expected performance of future progeny relative to the progeny of other animals. EPDs are one half of the Estimated Breeding Values (EBVs) of each animal and are predicted in mixed linear model analyses which incorporate numerator relationship matrices determined by pedigree information. EPDs are expressed in the units of measurement for the trait.

*Accuracy (ACC)*. The American Angus Association reports accuracy as *ACC* = 1 - $\sqrt{1-r_{TI}^{2}}$where ris squared correlation between predicted breeding value and true breeding value. These values were transformed in this study to obtain the rvalues necessary to obtain deregressed EBVs and weights for mixed model analyses.

*Birth Weight (BW)*. Birth weight in pounds of a bull’s progeny.

*Weaning Weight (WW)*. Weaning weight in pounds of progeny at ~305 d of age.

*Maternal Milk (MILK)*. Bull's genetic merit for the milk and mothering ability of his daughters. It is that part of a calf's weaning weight in pounds that is attributed to milk and mothering ability.

*Yearling Weight (YW)*. Weight in pounds of progeny at 12 months of age.

*Carcass Weight (CW)*. Hot carcass weight in pounds of progeny when slaughtered at ~15 mo of age.

*Mature Weight (MW)*. Mature weight in pounds of a bull’s daughters.

*Yearling Height (YH)*. Height in inches of a bull’s progeny measured at the hip at 12 months of age.

*Mature Height (MH)*. Mature height in inches of a bull's daughters measured at the hip.

*Fat Thickness (FAT)*. External fat thickness measured between the 12^th^ and 13^th^ ribs. Expressed in inches.

*Marbling (MARB)*. Intramuscular fat content of the *longissimus dorsi* muscle measured between the 12^th^ and 13^th^ ribs.

*Ribeye Muscle Area (RE)*. *Longissimus dorsi* cross-sectional area measured between the 12^th^ and 13^th^ ribs. Expressed in square inches.

*Calving Ease Direct (CED)*. Percentage of unassisted births, with a higher value indicating greater calving ease in first-calf females. It predicts the average ease with which a bull's calves will be born when he is bred to first-calf females.

*Calving Ease Maternal (CEM)*. Percentage of unassisted births with a higher value indicating greater calving ease in first-calf daughters. It predicts the average ease with which a bull's daughters will calve as first-calf heifers.

*Scrotal Circumference (SC)*. Bull’s scrotal circumference used as an indirect measure of female fertility. Expressed in centimeters.

*Heifer Pregnancy Rate (HP)*. Percentage of a bull’s daughters expected to become pregnant during a breeding season.

*Docility (DOC)*. Percentage differences between bulls’ progeny in temperament with higher values being more docile.

## Figure S1 – Manhattan plot of –log_10_(*p*-values) from the Poisson regression of genotypes coded as allele counts on birth date.

Red line corresponds to the Bonferroni corrected genome-wide significance line p = 1.12 × 10-6 and the blue line is genome-wide suggestive p = 1.0 × 10-4. Note the number of associations with highly inflated significance levels.

## Figure S2 – Q-Q plot of –log_10_(*p*-values) from the Poisson regression of genotypes coded as allele counts on birth date.

## Figure S3 – Deregressed calving ease direct EBV by birth date.

## Figure S4 – Deregressed yearling weight EBV by birth date.

## Figure S5 – Deregressed yearling height EBV by birth date.

## Figure S6 – Deregressed scrotal circumference EBV by birth date.

## Figure S7 – Deregressed docility EBV by birth date.

## Figure S8 – Deregressed heifer pregnancy EBV by birth date.

## Figure S9 – Deregressed calving ease maternal EBV by birth date.

## Figure S10 – Deregressed maternal milk EBV by birth date.

## Figure S11 – Deregressed mature weight EBV by birth date.

## Figure S12 – Deregressed mature height EBV by birth date.

## Figure S13 – Deregressed carcass weight EBV by birth date.

## Figure S14 – Deregressed marbling EBV by birth date.

## Figure S15 – Deregressed ribeye area EBV by birth date.

## Figure S16 – Deregressed fat thickness EBV by birth date.

## Figure S17 – Q-Q plot of –log_10_(*p*-values) for SNP effects estimated with EMMAX for Birth Date Selection Mapping.

**
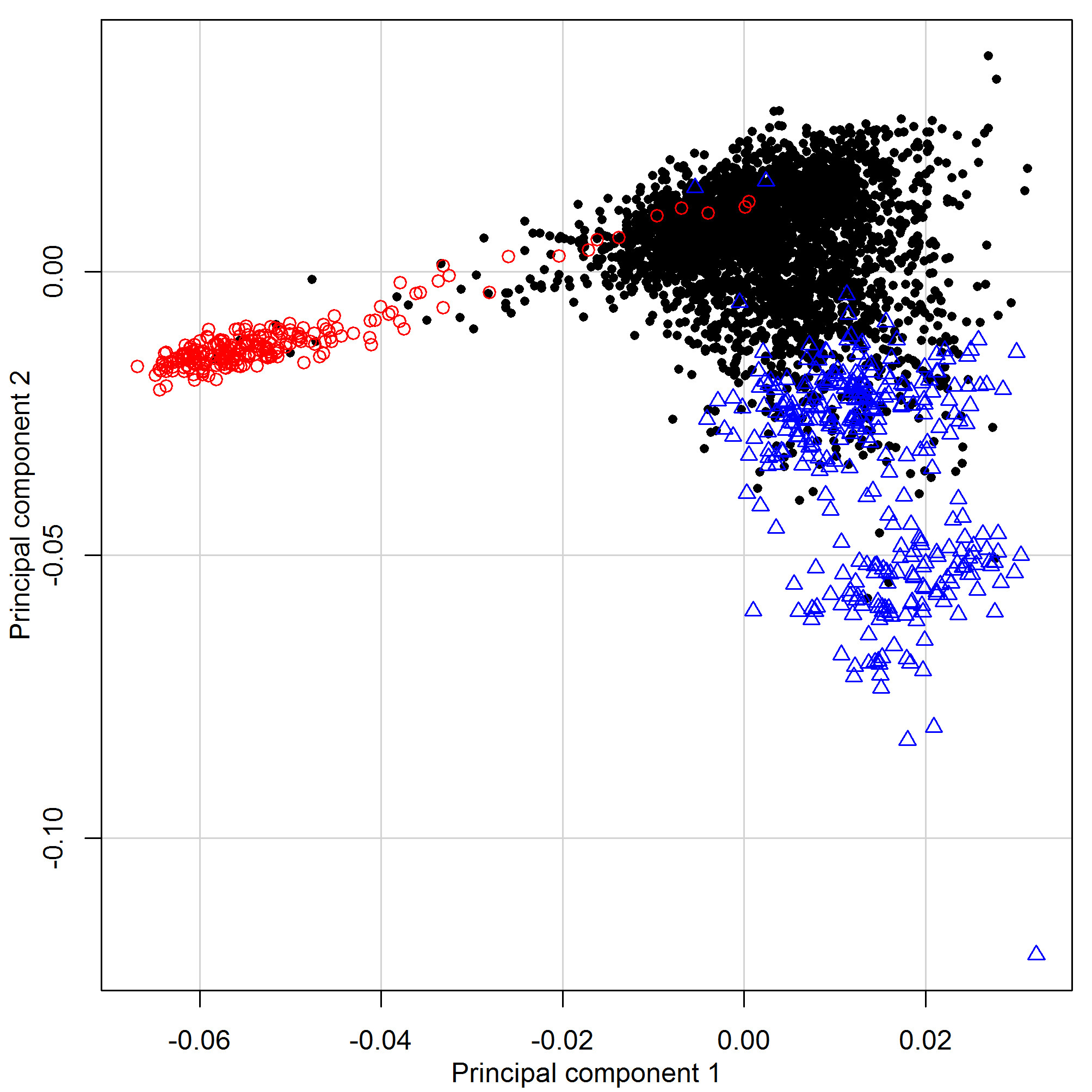
**

## Figure S18 – Principal component analysis of Angus AI sire genotypes.

From this analysis we identified two subgroups within our data. The first, denoted by red, is the Wye herd developed from imports from the British Isles and managed as a closed herd. The second is the rest of North American Angus. The blue triangles are a prominent AI sire (lower right corner), his sire, grandsire, progeny, and grandprogeny. Principal components 3 through 3,570 reveal family structure in a fashion similar to principal component 2. We correct for population structure and kinship by utilizing a genomic relationship matrix in our analyses of birth date.

## Figure S19 – Q-Q plots for of p-values from EMMAX analyses of reduced data subsets.

a. 1,237 animals from pedigree generations 58, 59, and 60.

b. 60 animals randomly sampled from pedigree generations 58, 59, and 60 (20/generation).

c. 1,237 animals randomly sampled from the entire data set.

d. 60 animals randomly sampled from the entire data set.

## Figure S20 – Manhattan plots for reduced data subsets.

a. 1,237 animals from generations 58, 59, and 60.

b. 60 animals randomly sampled from generations 58, 59, and 60 (20/generation).

c. 1,237 animals randomly sampled from the entire data set.

d. 60 animals randomly sampled from the entire data set.

## Figure S21 – Manhattan plot of SNP variances for calving ease direct.

## Figure S22 – Manhattan plot of SNP variances for birth weight.

## Figure S23 – Manhattan plot of SNP variances for yearling weight.

## Figure S24 – Manhattan plot of SNP variances for yearling height.

## Figure S25 – Manhattan plot of SNP variances for scrotal circumference.

## Figure S26 – Manhattan plot of SNP variances for docility.

## Figure S27 – Manhattan plot of SNP variances for heifer pregnancy.

## Figure S28 – Manhattan plot of SNP variances for calving ease maternal.

## Figure S29 – Manhattan plot of SNP variances for milk.

## Figure S30 – Manhattan plot of SNP variances for mature weight.

## Figure S31 – Manhattan plot of SNP variances for mature height.

## Figure S32 – Manhattan plot of SNP variances for carcass weight.

## Figure S33 – Manhattan plot of SNP variances for marbling.

## Figure S34 – Manhattan plot of SNP variances for ribeye area.

## Figure S35 – Manhattan plot of SNP variances for fat thickness.

## Table S1 – Regression of deregressed EBV on birth date for 16 production traits

| **Trait** | **Model Type** | **AIC** | **Adjusted* R^2^** | **Model p-value** | **Term** | **Estimate** | **Std. Error** | **T-value** | **p-value** |
| --- | --- | --- | --- | --- | --- | --- | --- | --- | --- |
| CED | Linear | 28124.62 | 0.0627 | <2.2e-16 | Int | -1113.0 | 76.3 | -14.59 | <2e-16 |
|  |  |  |  |  | BD | 0.5611 | 0.0382 | 14.70 | <2e-16 |
|  | Quadratic | 28120.17 | 0.0643 | <2.2e-16 | Int | 28375.6 | 11609.6 | 2.44 | 0.0146 |
|  |  |  |  |  | BD | -29.04 | 11.65 | -2.49 | 0.0128 |
|  |  |  |  |  | BD^2^ | 0.007426 | 0.002923 | 2.54 | 0.0111 |
| BW | Linear | 20751.91 | 0.0017 | 0.0125 | Int | 63.03 | 23.61 | 2.67 | 0.0076 |
|  |  |  |  |  | BD | -0.02952 | 0.01181 | -2.50 | 0.0125 |
|  | Quadratic | 20664.11 | 0.0286 | <2.2e-16 | Int | -33791.9 | 3549.5 | -9.52 | <2e-16 |
|  |  |  |  |  | BD | 33.95 | 3.56 | 9.53 | <2e-16 |
|  |  |  |  |  | BD^2^ | -0.008525 | 0.000894 | -9.54 | <2e-16 |
| WW | Linear | 32780.72 | 0.2909 | <2.2e-16 | Int | -5518.5 | 154.0 | -35.84 | <2e-16 |
|  |  |  |  |  | BD | 2.805 | 0.077 | 36.40 | <2e-16 |
|  | Quadratic | 32771.45 | 0.2931 | <2.2e-16 | Int | 73231.1 | 23447.4 | 3.12 | 0.0018 |
|  |  |  |  |  | BD | -76.23 | 23.53 | -3.24 | 0.0012 |
|  |  |  |  |  | BD^2^ | 0.01983 | 0.00590 | 3.36 | 0.0008 |
| YW | Linear | 31056.92 | 0.3085 | <2.2e-16 | Int | -9752.4 | 281.4 | -34.66 | <2e-16 |
|  |  |  |  |  | BD | 4.9598 | 0.1409 | 35.20 | <2e-16 |
|  | Quadratic | 31055.91 | 0.3090 | <2.2e-16 | Int | 64038.7 | 42599.6 | 1.50 | 0.1329 |
|  |  |  |  |  | BD | -69.13 | 42.77 | -1.62 | 0.1061 |
|  |  |  |  |  | BD^2^ | 0.01860 | 0.01074 | 1.73 | 0.0833 |
| YH | Linear | 7284.51 | -0.0003 | 0.5274 | Int | 4.4965 | 5.9446 | 0.76 | 0.4495 |
|  |  |  |  |  | BD | -0.001883 | 0.002978 | -0.63 | 0.5274 |
|  | Quadratic | 7221.19 | 0.0279 | 5.587e-15 | Int | -7278.5 | 895.2 | -8.13 | 6.97e-16 |
|  |  |  |  |  | BD | 0.315 | 0.899 | 8.13 | 6.81e-16 |
|  |  |  |  |  | BD^2^ | -0.001837 | 0.000226 | -8.14 | 6.69e-16 |

## Cont. Table S1 – Regression of deregressed EBV on birth date for 16 production traits

| **Trait** | **Model Type** | **AIC** | **Adjusted R^2^** | **Model p-value** | **Term** | **Estimate** | **Std. Error** | **T-value** | **p-value** |
| --- | --- | --- | --- | --- | --- | --- | --- | --- | --- |
| SC | Linear | 9875.76 | 0.0548 | <2.2e-16 | Int | -99.82 | 8.34 | -11.96 | <2e-16 |
|  |  |  |  |  | BD | 0.0503 | 0.0042 | 12.03 | <2e-16 |
|  | Quadratic | 9873.41 | 0.0561 | <2.2e-16 | Int | 2556.3 | 1273.2 | 2.01 | 0.0448 |
|  |  |  |  |  | BD | -2.617 | 1.278 | -2.05 | 0.0408 |
|  |  |  |  |  | BD^2^ | 0.000670 | 0.000321 | 2.09 | 0.0371 |
| DOC | Linear | 14425.05 | 0.0079 | 0.0006 | Int | -1202.2 | 352.6 | -11.96 | 0.0007 |
|  |  |  |  |  | BD | 0.6095 | 0.1765 | 3.45 | 0.0006 |
|  | Quadratic | 14426.88 | 0.0073 | 0.0024 | Int | 23515.7 | 59954.3 | 0.39 | 0.6950 |
|  |  |  |  |  | BD | -24.19 | 60.14 | -0.40 | 0.6876 |
|  |  |  |  |  | BD^2^ | 0.006218 | 0.015080 | 0.41 | 0.6802 |
| HP | Linear | 6261.80 | 0.0029 | 0.0821 | Int | 455.72 | 252.83 | 1.80 | 0.0719 |
|  |  |  |  |  | BD | -0.2205 | 0.1267 | -1.74 | 0.0821 |
|  | Quadratic | 6263.68 | 0.0016 | 0.2087 | Int | -17220.1 | 52512.4 | -0.33 | 0.7431 |
|  |  |  |  |  | BD | 17.52 | 52.70 | 0.33 | 0.7397 |
|  |  |  |  |  | BD^2^ | -0.004451 | 0.013220 | -0.34 | 0.7365 |
| CEM | Linear | 17952.65 | 0.0436 | <2.2e-16 | Int | -1167.0 | 123.9 | -9.42 | <2e-16 |
|  |  |  |  |  | BD | 0.5912 | 0.0621 | 9.52 | <2e-16 |
|  | Quadratic | 17951.18 | 0.0448 | <2.2e-16 | Int | -38160.0 | 19856.5 | -1.92 | 0.0548 |
|  |  |  |  |  | BD | 37.79 | 19.97 | 1.89 | 0.0586 |
|  |  |  |  |  | BD^2^ | -0.00935 | 0.00502 | -1.86 | 0.0626 |
| MILK | Linear | 19571.33 | 0.1602 | <2.2e-16 | Int | -2819.6 | 143.6 | -19.64 | <2e-16 |
|  |  |  |  |  | BD | 1.4304 | 0.0720 | 19.88 | <2e-16 |
|  | Quadratic | 19572.43 | 0.1601 | <2.2e-16 | Int | -24577.3 | 22918.4 | -1.07 | 0.2837 |
|  |  |  |  |  | BD | 23.31 | 23.04 | 1.01 | 0.3119 |
|  |  |  |  |  | BD^2^ | -0.005498 | 0.005791 | -0.95 | 0.3425 |

## Cont. Table S1 – Regression of deregressed EBV on birth date for 16 production traits

| **Trait** | **Model Type** | **AIC** | **Adjusted R^2^** | **Model p-value** | **Term** | **Estimate** | **Std. Error** | **T-value** | **p-value** |
| --- | --- | --- | --- | --- | --- | --- | --- | --- | --- |
| MW | Linear | 16702.55 | 0.0114 | 5.999e-05 | Int | -3729.2 | 943.0 | -3.96 | 8.08e-05 |
|  |  |  |  |  | BD | 1.9039 | 0.4729 | 4.03 | 6.00e-05 |
|  | Quadratic | 16672.11 | 0.0346 | 2.998e-11 | Int | -876824.0 | 152533.8 | -5.75 | 1.12e-08 |
|  |  |  |  |  | BD | 879.5 | 153.3 | 5.74 | 1.20e-08 |
|  |  |  |  |  | BD^2^ | -0.2205 | 0.0385 | -5.72 | 1.29e-08 |
| MH | Linear | 5752.40 | 0.0072 | 0.0013 | Int | -50.52 | 16.01 | -3.16 | 0.0016 |
|  |  |  |  |  | BD | 0.0259 | 0.0080 | 3.22 | 0.0013 |
|  | Quadratic | 5724.30 | 0.0294 | 1.709e-09 | Int | -14675.8 | 2653.3 | -5.53 | 3.85e-08 |
|  |  |  |  |  | BD | 14.73 | 2.67 | 5.52 | 4.05e-08 |
|  |  |  |  |  | BD^2^ | -0.003695 | 0.000670 | -5.51 | 4.27e-08 |
| CW | Linear | 28587.12 | 0.0658 | <2.2e-16 | Int | -5169.4 | 394.3 | -13.11 | <2e-16 |
|  |  |  |  |  | BD | 2.6028 | 0.1974 | 13.19 | <2e-16 |
|  | Quadratic | 28585.05 | 0.0669 | <2.2e-16 | Int | 112383.2 | 58298.6 | 1.93 | 0.0540 |
|  |  |  |  |  | BD | -115.4 | 58.5 | -1.97 | 0.0487 |
|  |  |  |  |  | BD^2^ | 0.0296 | 0.0147 | 2.02 | 0.0439 |
| MARB | Linear | 9903.56 | 0.0389 | <2.2e-16 | Int | -53.54 | 4.72 | -11.35 | <2e-16 |
|  |  |  |  |  | BD | 0.0271 | 0.0024 | 11.48 | <2e-16 |
|  | Quadratic | 9905.41 | 0.0386 | <2.2e-16 | Int | 223.7 | 716.3 | 0.31 | 0.7548 |
|  |  |  |  |  | BD | -0.2510 | 0.7186 | -0.35 | 0.7269 |
|  |  |  |  |  | BD^2^ | 0.000070 | 0.000180 | 0.39 | 0.6988 |
| RE | Linear | 9365.74 | 0.0356 | <2.2e-16 | Int | -46.58 | 4.24 | -10.98 | <2e-16 |
|  |  |  |  |  | BD | 0.0234 | 0.0021 | 11.02 | <2e-16 |
|  | Quadratic | 9367.72 | 0.0353 | <2.2e-16 | Int | 53.95 | 646.5 | 0.08 | 0.9335 |
|  |  |  |  |  | BD | -0.0775 | 0.6485 | -0.12 | 0.9049 |
|  |  |  |  |  | BD^2^ | 0.000025 | 0.000163 | 0.16 | 0.8764 |

## Cont. Table S1 – Regression of deregressed EBV on birth date for 16 production traits

| **Trait** | **Model Type** | **AIC** | **Adjusted R^2^** | **Model p-value** | **Term** | **Estimate** | **Std. Error** | **T-value** | **p-value** |
| --- | --- | --- | --- | --- | --- | --- | --- | --- | --- |
| FAT | Linear | -2720.33 | 0.0074 | 7.217e-07 | Int | -3.32 | 0.67 | -4.93 | 8.81e-07 |
|  |  |  |  |  | BD | 0.001676 | 0.000338 | 4.97 | 7.22e-07 |
|  | Quadratic | -2732.61 | 0.0115 | 3.733e-09 | Int | 381.9 | 101.9 | 3.75 | 0.0002 |
|  |  |  |  |  | BD | -0.3848 | 0.1022 | -3.76 | 0.0002 |
|  |  |  |  |  | BD^2^ | 0.000097 | 0.000026 | 3.78 | 0.0002 |

*****Adjusted for the number of terms in the model.

## Table S2 – Relative selection intensities for 16 production traits estimated from the regression of the top 935 birth date ASEs on standardized SNP ASE coefficients.

After pruning SNPs in complete LD, the 935 SNPs with the largest birth date variance were fit in the model. ASEs were standardized by conversion to coefficients of *pqASE*/σ*_ASE_*. Each trait was fit in an individual regression. The *F*-statistic degrees of freedom for the models were 2 and 933.

| **Trait** | **Adjusted *R^2^*** | ***F* statistic** | **Model p-value** | **Term** | **Estimate** | **Est. Standard Error** | **T-statistic** | **p-value** |
| --- | --- | --- | --- | --- | --- | --- | --- | --- |
| BW | -0.0010 | 0.046 | 0.955 | Int | 0.3861 | 0.0031 | 124.250 | <1e-267 |
|  |  |  |  | BW | -1.7628 | 0.0395 | 44.674 | 5.50e-234 |
| WW | 0.6168 | 752.180 | 2.83e-195 | Int | 0.1958 | 0.0021 | 94.773 | <1e-267 |
|  |  |  |  | WW | 6.3225 | 0.0150 | 422.310 | <1e-267 |
| Milk | 0.3338 | 234.502 | 3.10e-83 | Int | 0.0625 | 0.0025 | 24.693 | 5.35e-104 |
|  |  |  |  | Milk | 5.3888 | 0.0255 | 211.159 | <1e-267 |
| YW | 0.5142 | 494.770 | 3.33e-147 | Int | 0.0838 | 0.0021 | 39.375 | 1.53e-200 |
|  |  |  |  | YW | 5.5263 | 0.0157 | 352.792 | <1e-267 |
| YH | 0.0060 | 3.308 | 0.037 | Int | 0.3962 | 0.0030 | 129.902 | <1e-267 |
|  |  |  |  | YH | 0.3720 | 0.0442 | 8.414 | 1.48e-16 |
| CWT | 0.2740 | 176.775 | 7.99e-66 | Int | 0.3720 | 0.0029 | 127.458 | <1e-267 |
|  |  |  |  | CWT | 6.4494 | 0.0347 | 185.969 | <1e-267 |
| MARB | 0.2842 | 185.881 | 1.13e-68 | Int | 0.0665 | 0.0033 | 20.070 | 9.28e-75 |
|  |  |  |  | MARB | 8.5703 | 0.0399 | 214.813 | <1e-267 |
| REA | 0.1829 | 105.025 | 7.28e-42 | Int | -0.0053 | 0.0031 | 1.737 | 0.083 |
|  |  |  |  | REA | 6.1111 | 0.0366 | 167.027 | <1e-267 |
| FT | 0.0235 | 11.726 | 9.35e-06 | Int | 0.2799 | 0.0030 | 92.555 | <1e-267 |
|  |  |  |  | FT | 1.6251 | 0.0438 | 37.103 | 7.75e-186 |
| MWT | 0.0903 | 46.846 | 4.10e-20 | Int | 0.2185 | 0.0033 | 66.851 | <1e-267 |
|  |  |  |  | MWT | 6.0119 | 0.0444 | 135.294 | <1e-267 |
| MHT | 0.0762 | 39.043 | 5.20e-17 | Int | 0.0977 | 0.0035 | 28.018 | 7.88e-126 |
|  |  |  |  | MHT | 7.3312 | 0.0506 | 144.792 | <1e-267 |
| SC | 0.1508 | 83.409 | 4.71e-34 | Int | 0.0706 | 0.0030 | 23.824 | 2.26e-98 |
|  |  |  |  | SC | 5.3135 | 0.0412 | 128.934 | <1e-267 |

## Table S2 continued.

| **Trait** | **Adjusted *R^2^*** | ***F* statistic** | **Model p-value** | **Term** | **Estimate** | **Est. Standard Error** | **T-statistic** | **p-value** |
| --- | --- | --- | --- | --- | --- | --- | --- | --- |
| CED | 0.2309 | 140.730 | 3.85e-54 | intercept | 0.1591 | 0.0032 | 50.166 | 3.67e-267 |
|  |  |  |  | CED | 7.4788 | 0.0401 | 186.414 | <1e-267 |
| CEM | 0.2848 | 186.449 | 7.56e-69 | intercept | 0.3092 | 0.0028 | 109.189 | <1e-267 |
|  |  |  |  | CEM | 6.5138 | 0.0351 | 185.455 | <1e-267 |
| HP | 0.0243 | 12.141 | 6.23e-06 | intercept | 0.3849 | 0.0030 | 127.575 | <1e-267 |
|  |  |  |  | HP | 1.6541 | 0.0492 | 33.629 | 4.98e-163 |
| DOC | -0.0006 | 0.226 | 0.797 | intercept | 0.4223 | 0.0031 | 136.211 | <1e-267 |
|  |  |  |  | DOC | 1.4784 | 0.0438 | 33.784 | 4.70e-164 |
